# High-drug-loading capacity of redox-activated biodegradable nanoplatform for active targeted delivery of chemotherapeutic drugs

**DOI:** 10.1093/rb/rbaa027

**Published:** 2020-07-06

**Authors:** Hai Zhang, Jianqin Yan, Heng Mei, Shengsheng Cai, Sai Li, Furong Cheng, Jun Cao, Bin He

**Affiliations:** r1 School of Chemical Engineering, Sichuan University, No.24 South Section 1, Yihuan Road, Chengdu 610065, China; r2 National Engineering Research Center for Biomaterials, Sichuan University, No.29 Wangjiang Road, Chengdu 610064, China

**Keywords:** good stability, π-conjugated moieties, active-targeting, redox-activated

## Abstract

Challenges associated with low-drug-loading capacity, lack of active targeting of tumor cells and unspecific drug release of nanocarriers synchronously plague the success of cancer therapy. Herein, we constructed active-targeting, redox-activated polymeric micelles (HPGssML) self-assembled aptamer-decorated, amphiphilic biodegradable poly (benzyl malolactonate-co-ε-caprolactone) copolymer with disulfide linkage and π-conjugated moieties. HPGssML with a homogenous spherical shape and nanosized diameter (∼150 nm) formed a low critical micellar concentration (10^−3 ^mg/mL), suggesting good stability of polymeric micelles. The anticancer drug, doxorubicin (DOX), can be efficiently loaded into the core of micelles with high-drug-loading content via strong π–π interaction, which was verified by a decrease in fluorescence intensity and redshift in UV adsorption of DOX in micelles. The redox sensitivity of polymeric micelles was confirmed by size change and *in vitro* drug release in a reducing environment. Confocal microscopy and flow cytometry assay demonstrated that conjugating aptamers could enhance specific uptake of HPGssML by cancer cells. An *in vitro* cytotoxicity study showed that the half-maximal inhibitory concentration (IC_50_) of DOX-loaded HPGssML was two times lower than that of the control group, demonstrating improved antitumor efficacy. Therefore, the multifunctional biodegradable polymeric micelles can be exploited as a desirable drug carrier for effective cancer treatment.

## Introduction

Cancer is one of the major diseases which threatens human beings in the world today [1]. Despite the development of various innovative therapy methods, chemotherapy is still the first-line treatment for cancer [2, 3]. However, the therapeutic efficacy of chemotherapeutic drugs, such as doxorubicin (DOX), in the free formulation is limited by poor solubility and low bioavailability as well as by their nonselective targeting ability [4, 5]. Although nanoscale drug delivery systems (DDSs) hold great promise in addressing drawbacks and enhancing antitumor efficacy, such as the utilization of enhanced permeability and retention effect on passive delivery of anticancer drugs to tumor site [4, 6–8], the current DDSs face challenges associated with low-drug-loading capacity, lack of active targeting and slow drug release, which significantly compromise antitumor efficacy [9].

Many strategies have been utilized to improve drug loading capacity, including variations of hydrophobic architecture [10–13], the formation of dimer drugs [10, 11] and an increase in interactions between the drug and the inner core of nanocarriers (stereocomplexation) [12], host–guest interaction [13, 14], π– π stacking interaction [15, 16]. Inspired by the structure of most anticancer drugs (e.g. DOX, paclitaxel and camptothecine) with complex aromatic π–π conjugated structure, the increase of π–π stacking interaction between anticancer drugs and polymeric micelles is favorable for drug loading. In our previous study [17], small molecules with π-conjugated architecture on the terminal group of methoxy poly(ethylene glycol)-poly(ε-caprolactone) (mPEG-PCL) micelles were immobilized. The results showed almost two times higher drug loading content (DLC) was obtained compared with traditional mPEG-PCL micelles. However, limited π-conjugated moieties in polymeric micelles and low reaction efficiency of conjugated small molecules on the polymer were needed for further improvement. β-Benzyl ester malic acid lactone (β-MLABz) is a side-protected malic acid lactone monomer. It is generally used as a monomer in the synthesis of biodegradable poly(β-benzyl ester malate) (PMLABz) via ring-opening polymerization [18]. Most studies have focused on constructing intelligent polymeric micelles through conjugating various functional molecules with carboxyl groups on the side chains of poly(β-malic acid) after deprotection [19, 20]. Notably, a large number of π-conjugated on the pendant of PMLABz enhances drug loading capacity through π–π stacking interaction and further improves the stability of polymeric micelles, which has not been previously reported to the best of our knowledge.

Active targeting delivery of drug-loaded polymeric micelles to tumor sites is a prospective approach to improve therapeutic efficacy and reduce undesired effects associated with chemotherapeutic drugs [21–24]. The use of recognition motifs, such as folic acid [21], transferrin [22], peptides [23, 24] and antibodies [25] for specific tumor active targeting delivery is the most commonly used strategy. Compared with other recognition motifs, aptamers are single-stranded oligonucleotides that can interact with the targeting ligands of cells, with selective, high affinity. Aptamer A8, an 8-amino acid (SPWPRPTY) peptide-aptamer, can specifically bind to the extracellular domain of membraned HSP70, which is abundantly expressed on cancer cells. Accumulating evidence validated that conjugation of Aptamer A8 on polymeric micelles can achieve efficient tumor targeting and improve internalization efficiency [23, 24]. Considering that aptamers possess outstanding targeting ability, it is a promising recognition motif for active targeted delivery of polymeric micelles to tumor sites.

Stimuli-sensitive polymeric micelles are capable of releasing drugs site specifically, responding to environmental changes between tumor microenvironments and healthy tissues [26], for example, changes in enzyme [27], pH [28] and glutathione (GSH) [29, 30] levels. Appropriate drug release can significantly improve therapeutic efficacy. For instance, the presence of a high level of GSH (2–10 mM) in tumor cells, compared with that of healthy tissues, proves that the disulfide bond can be easily cleaved by intracellular GSH while remaining stable in physicochemical conditions [31]. Therefore, taking the significant discrepancies in GSH distribution in tumor cells and healthy cells, disulfide bonds can be introduced into polymeric micelles for GSH-sensitive drug release.

**Scheme 1:**
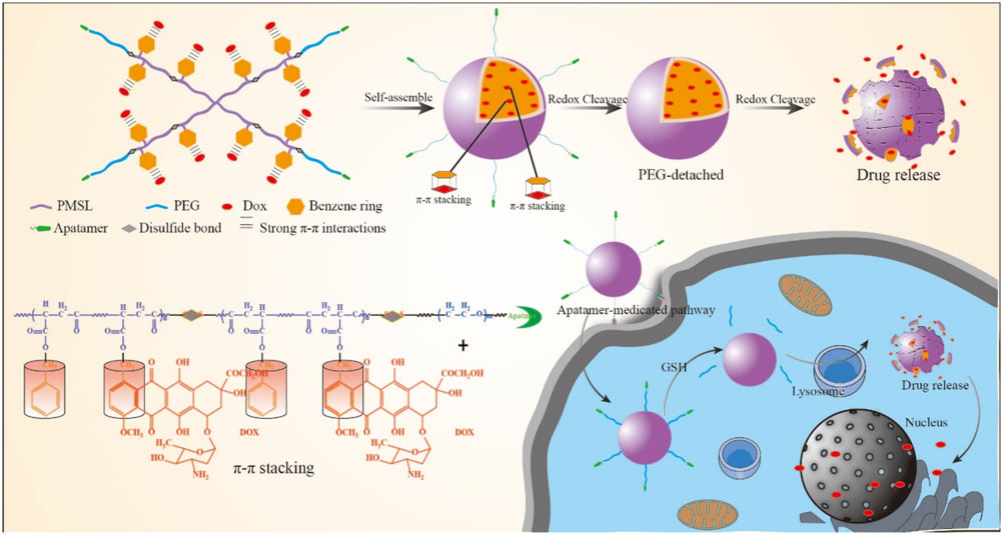
The aptamer-decorated, reduction-sensitive polymeric micelles self-assembled biodegradable polyester-based amphiphilic copolymer and the concept for a proposed behavior of polymeric micelles for anticancer drug loading, delivery and release.

In this work, we attempted to develop active-targeting, redox-activated polymeric micelles (HPGssML) self-assembled aptamer-decorated, biodegradable poly (benzyl malolactonate-co-ε-caprolactone) copolymer with disulfide linkage and π-conjugated moieties to load anticancer drug DOX via strong π–π interaction for cancer therapy (Scheme 1). The multifunctional biodegradable copolymers were composed of aptamer-modified PEG as the hydrophilic segment and poly(benzyl malolactonate-ε-caprolactone) (PMSL) with π-conjugated moieties as the hydrophobic segment. Methoxy poly(ethylene glycol)-PMSL was used as a control. The physicochemical properties of the constructed polymeric micelles, including size, morphology and drug loading capacity, were investigated. The *in vitro* drug release, cellular uptake and antitumor efficiency of DOX-loaded polymeric micelles were thoroughly studied.

## Materials and methods

### Materials

Methoxy poly(ethylene glycol) (mPEG, *M*_w_ = 2000 g/mol), poly(ethylene glycol) (OH–PEG–OH, *M*_w_ = 2000 g/mol), benzyl malolactonate, Tin(II) 2-ethylhexanote (Sn(Oct)_2_), bis(2-carboxyethyl) disulfide and 4-dimethyl aminopyridine were purchased from Sigma-Aldrich. 6-maleiMidocaproic acid (98%), 2,2′-dithiodipropionic acid (98%) were bought from Tokyo Chemical Industry (TCI, Shanghai). *N,N*-dicyclohexyl carbodiimide (DCC) and succinic anhydride were purchased from heowns. Doxorubicin hydrochloride (DOX · HCl; Zhejiang Hisun Pharmaceutical, China) was deprotonated according to the previously reported method [32]. Aptamer A8, 8-amino acid (CSPWPRPTY-SH) peptide-aptamer (HSPs), was obtained from GL Biochem Ltd (Shanghai). Dialysis membranes (molecular weight cutoff [MWCO] = 1000, 2000 and 3500 Da) were commercially available from Spectrum/Por (Houston, TX, USA). Dulbecco’s Modified Eagle’s Medium (DMEM) and Roswell Park Memorial Institute 1640 medium (RPMI1640), fetal bovine serum, penicillin–streptomycin were ordered from Life Technologies Corporation (Gibco, USA) and directly used for cells test. All the other chemicals and solvents were purchased from Kelong Chemical Co. (Chengdu, China) and used without purification.

### Characterizations

The ^1^H NMR spectra were performed on Brucker Avance II NMR spectrometer at 400 MHz using CDCl_3_, (CD_3_)_2_SO as solvents with 0.5% tetramethylsilane as the internal standard. The size and size distribution of polymeric micelles were carried out using a dynamic light scattering (DLS, Malvern Zetasizer Nano ZS). Scanning electron microscopy was used to observe the morphology and particle size of micelles. The π–π interaction between DOX and polymeric micelles was determined by UV-vis absorption (Specord 200 PLUS) and Fluorescence spectra (HITACHIF-700).

### Synthesis of PMSL

The synthesis of benzyl malolactonate (BMA) monomer was the same as the procedure previously reported [18]. PMSL copolymer was synthesized by ring-opening polymerization of ε-caprolactone and BMA in the presence of Sn(Oct)_2_ (mass ratio 1/1000) as a catalyst and 2-hydroxyethyl disulfide as an initiator. In a typical procedure, ε-caprolactone (1.522 g, 13.4 mmol), BMA (1.3886 g, 6.7 mmol) and 2-hydroxyethyl disulfide initiator (0.101 g, 0.445 mmol) were put into a dry polymerization tube. Then Sn (Oct)_2_ in dry toluene was added. Under the condition of vacuum, the tube was sealed and put into an oil bath at 120°C for 48 h. After that, the PMSL was dissolved in dichloromethane (CH_2_Cl_2_) and precipitated in diethyl ether. The final white powder was filtered and vacuum dried at room temperature. The structure of the polymer was characterized by the ^1^H NMR spectrum.

### Synthesis of functional PEG (Mal-PEG-ss-COOH)

6-Maleimidocaproic acid (0.213 g, 1 mmol), DMAP (0.012 g, 0.1 mmol) and HO–PEG–OH (2 g, 1 mmol) were dissolved in anhydrous CH_2_Cl_2_ under nitrogen atmosphere. A solution of DCC (4 g, 10 mmol) in tetrahydrofuran (THF) was added dropwise into the mixture and stirred at room temperature for 48 h. The solid white precipitate was filtrated, and then the filtrate was condensed and precipitated in a large amount of diethyl ether. After precipitated in cold diethyl ether, the product (Mal-PEG-OH) was vacuum dried at room temperature. Mal-PEG-OH (1.1 g, 0.5 mmol), DMAP (0.024 g, 0.2 mmol) and Bis(2-carboxyethyl) disulfide (0.21 g, 1 mmol) were dissolved in anhydrous tetrahydrofuran under nitrogen atmosphere. Then DCC (4 g, 20 mmol) dissolved in THF was added dropwise into the mixture. The mixture was stirred at room temperature for 48 h. The solid white precipitate was filtrated, condensed and precipitated in a large amount of diethyl ether. The product (Mal-PEG-ss-COOH) was vacuum dried at room temperature. The structure of the polymer was characterized by the ^1^H NMR spectrum.

### Synthesis of multifunctional biodegradable copolymer (HPGssML)

Mal-PEG-ss-COOH (0.5 g, 0.21 mmol), DMAP (0.004 g, 0.03 mmol) and PMSL (0.8 g, 0.21 mmol) were dissolved in anhydrous CH_2_Cl_2_ under nitrogen atmosphere. Then DCC (1.3 g, 6.3 mmol) dissolved in dichloromethane (DCM) was slowly added into the mixture. The solution was stirred at room temperature for 48 h. The white precipitate was filtrated, and then the filtrate was condensed and precipitated in cold diethyl ether. One gram of Mal-PEG-ss-PMSL and 0.2 g of CSPWPRPTY-SH were dissolved in dimethyl sulfoxide (DMSO) and stirred at room temperature for 1 day. Then the solution was diluted with deionized water and transferred to a dialysis tubing. The solution was dialyzed against deionized water for 24 h. The outer phase was replaced with deionized water every 4 h. The solution in the dialysis tubing was centrifuged and lyophilized. The final white powder product was obtained. The detailed synthesis procedure of mPEG-PMSL (PGML) was exhibited in Supplementary Data. The structures of polymers were characterized by the ^1^H NMR spectrum.

### Critical micellar concentration measurement

The critical micellar concentrations (CMCs) of PGML and HPGssML amphiphilic copolymers were determined by fluorescence spectra (F-7000, Hitachi Co., Japan) using pyrene as a probe. The pyrene aqueous solution with a concentration of 6.0 × 10^−7^ M was prepared. Afterward, the polymeric micellar solution was diluted by a pyrene aqueous solution to the final concentration of 3 × 10^−8^ g/L to 0.5 g/L. Then the diluted solution was detected by fluorescence to determine their CMCs. The emission wavelength was fixed at 390 nm. The fluorescent intensity at 334 and 338 nm was recorded. The value (I_338_/I_334_) was calculated and plotted against the logarithm of amphiphilic copolymers concentration.

### Preparation of drug-loaded polymeric micelles

The amphiphilic copolymer (HPGssML, 10 mg) and DOX (2.5 mg) were dissolved in 1 mL of DMSO and stirred for 1 h. Then the solution was dropped into 10 mL of deionized water with vigorous stirring. After stirring for 6 h, the mixture was transferred into dialysis tubing (Spectra/Por MWCO = 2000) and dialyzed against deionized water at room temperature for 8 h. The unloaded drug was removed by centrifugation (3000 r/min, 5 min) and the drug-loaded micelles were lyophilized. Afterward, DOX-loaded HPGssML were obtained. PGML micelles were obtained via the same procedure in which PGML copolymer was replaced by HPGssML copolymer. The whole procedure was worked in the dark. The preparation of PGML or HPGssML was similar to that of DOX-loaded HPGssML. The difference was that DOX was not added to the mixture. The DLC and encapsulation efficiency were determined by UV-vis measurement (maximum absorption wavelength of DOX at 480 nm). DLC and drug loading efficiency were calculated according to the following formulas:

(1) DLC (%)=(weight of loaded drug/weight of drug−loaded polymeric micelles)×100%,

(2) EE (%)=(weight of loaded drug/weight of drug in feeding)×100%.

### Redox-responsive behaviors of polymeric micelles

To investigate the redox-responsive behaviors, polymeric micelles were incubated with different biomimetic solutions (pH 7.4, pH 7.4 with 0.5 mM or 10 mM GSH) at 37°C, respectively. The size and size distribution of micelles were measured at designed intervals by DLS (Malvern ZetasizerNano ZS).

The drug release behaviors of DOX-loaded PGML and HPGssML were detected in different biomimetic solutions (pH 7.4, pH 5.0 and pH 5.0 with 10 mM GSH, ionic strength = 0.01 M). First, 1 mL of polymeric micellar solution was added into dialysis tubing (Spectra/Por MWCO = 1000) and immersed in vials containing 25 mL of corresponding release media. Afterward, the vials were placed in a shaking bed at 37°C. At prescribed time intervals, 1 mL of released media was taken out and the same volume of freshly released media with the corresponding pH was added into the vials. The amount of released DOX was detected by a fluorescence spectrometer. The excitation wavelength of DOX was designed at 480 nm with the emission wavelength at 550 nm. The release experiments were done under sink conditions. The release experiments were conducted in triplicate, and the results were demonstrated as mean ± SD.

### Cytotoxicity assay

The cytotoxicity of PGML or HPGssML was tested using the 3-(4,5-dimethylthiazol-2-yl)-2,5-diphenyltetrazolium bromide (MTT) assay against L929 mouse fibroblast cells, mouse breast cancer cells (4T1) and human breast cancer cells (MDA-MB-231) which were purchased from the Chinese Academy of Sciences Cell Bank. After those cells were seeded in 96-well plates, respectively, with 4 × 10^4^ cells/well in 100 μL of medium for 24 h, the medium was removed and replaced with 100 μL of medium containing different concentrations of PGML (10–300 μg/mL) or HPGssML (10–300 μg/mL) micelles. For 48 h incubation, the medium was removed and the wells were washed with phosphate buffered solution (PBS) (pH 7.4). Then, 10 μL of 5 mg/mL MTT solution in cell culture media was added to each well. After incubation for 4 h, DMSO (100 μL) was added in each well. The absorbance was measured in a Thermo Scientific MK3 at the wavelength of 490 nm.

### Cellular uptake study

The cellular uptake behaviors of micelles against 4T1 cells, MDA-MB-231 cells and MCF-7 cells were studied by confocal laser scanning microscopy (CLSM) and flow cytometry tests. 4T1, MDA-MB-231 and MCF-7 cells at a logarithm phase were seeded on glass dishes (diameter = 35 mm) for 24 h. Then, 1 mL of DOX · HCl and DOX-loaded micelles in DMEM (DOX = 10 μg/mL) was added into dishes. After incubated for 2 and 4 h at 37°C, respectively, the medium was removed, and the dishes were rinsed with PBS (pH = 7.4). The cell nuclei were stained with DAPI for 5 min, and the culture medium was replaced with PBS. The cells were imaged by CLSM (TCP SP5, Leica, Germany) and the excited/emission wavelength was designed as 488/550 nm, respectively.

For the flow cytometry tests, MCF-7 cells were seeded in 6-well plates at a density of 1 × 10^6^ cells/well and incubated for 24 h. The DOX-loaded micelles were added into the plates with the DOX concentration of 10 µg/mL for 2 and 4 h. The culture medium was eliminated, and the cells were washed with PBS and harvested by trypsinization. Finally, the cells were resuspended in PBS after centrifugation (1000 rpm, 5 min) and the fluorescence intensity was measured on a BD FACS Calibur flow cytometer (Beckton Dickinson).

### *In vitro* anticancer activity

The *in vitro* anticancer activity was carried out in 4T1, MCF-7 and MDA-MB-231. Those cells were seeded in 96-well plates at a density of 1 × 10^4^ per well for 24 h, respectively. The culture medium was removed and replaced with fresh medium containing drug-loaded micelles with different DOX concentrations. After incubated for 48 h at 37°C, the culture medium was removed, and the wells were rinsed with PBS (pH = 7.4). Thereafter, MTT in PBS (5 mg/mL, 10 μL) was added to each well for another 4 h. The medium was carefully removed and DMSO (100 μL) was added in each well. The absorbance was measured in a Thermo Scientific MK3 (Thermo Fisher, USA) at the wavelength of 492 nm.

## Results and discussion

### Synthesis and characterization of multifunctional copolymer

**Scheme 2:**
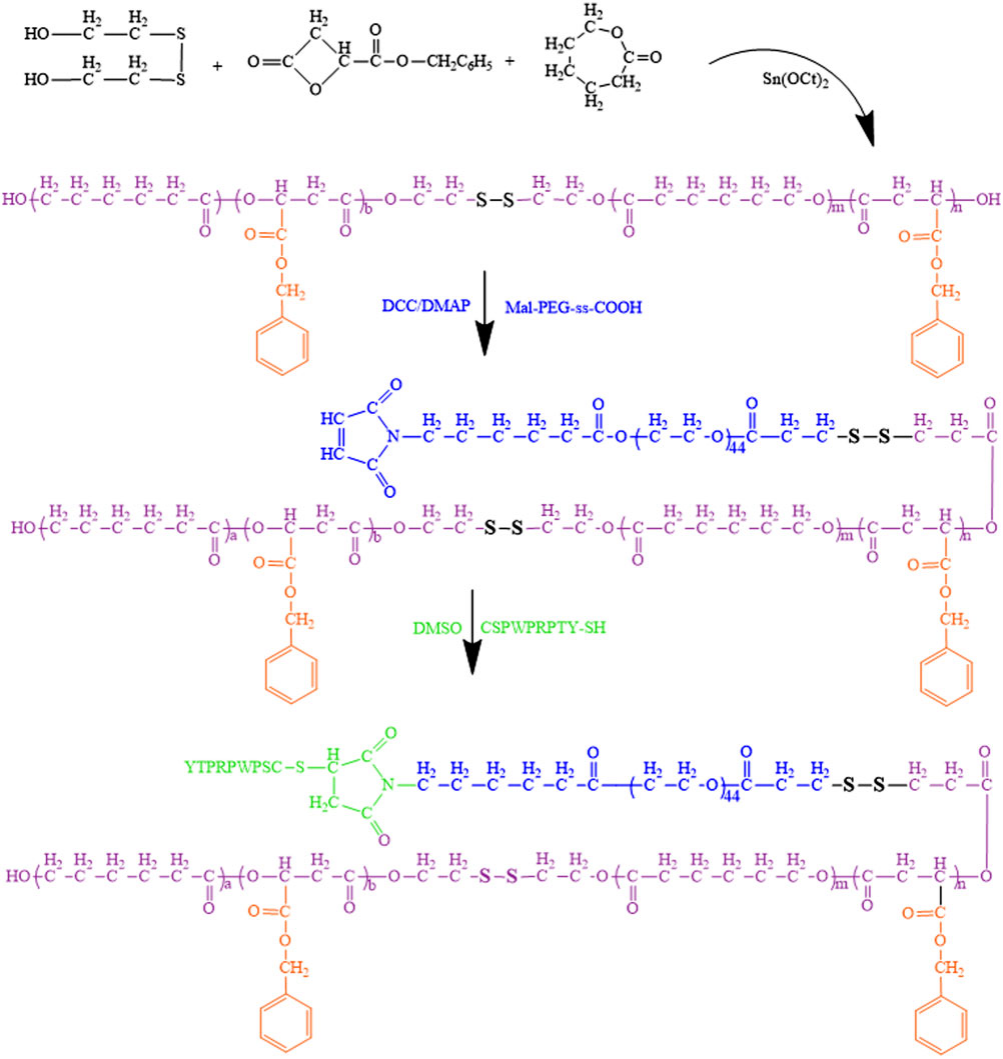
The synthetic routes of HPGssML copolymers.

**Figure 1:**
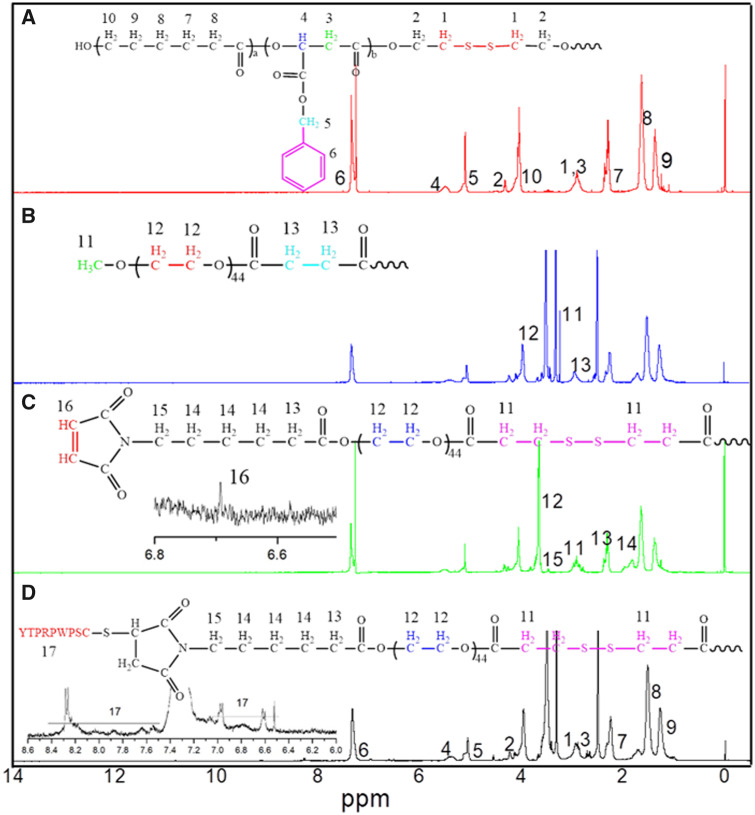
The ^1^H NMR spectra of (**A**) PMSL, (**B**) PGML, (**C**) Mal-PEG-ss-PMSL and (**D**) HPGssML. The solvents of PMSL and Mal-PEG-ss-PMSL were CDCl_3_. The solvents of PGML and HPGssML were (CD_3_)_2_SO.

The synthetic routes of BMA monomer, functional PEG, PGML and HPGssML are illustrated in Scheme 2 and Supplementary Schemes S1 and S2.The chemical structures of copolymers in each step were characterized by ^1^H NMR (Fig. 1 and Supplementary Fig. S1). The characteristic proton peaks at ∼3.6 and ∼4.8 ppm attributed to the methylene of *b*-BMA moiety confirmed the successful preparation of BMA monomer (Supplementary Fig. S1A). The structures of modified PEG (mPEG-COOH, Mal-PEG-OH and Mal-PEG-ss-COOH) were all demonstrated by ^1^H NMR (Supplementary Fig. S1B–D) respectively. PMSL copolymer backbone was synthesized by ring opening polymerization. Fig. 1A showed that all the proton signals attributed to PMSL blocks emerged, including the peaks at *δ* = 4.2, 2.2–2.5 and 1.5 ppm attributed to –CH_2_ protons of ε-caprolactone repeated units, respectively, and the signals at *δ* = 5.5 and 2.7–3.1 ppm assigned to the –CH and –CH_2_ protons of BMA repeated units in the copolymer. Additionally, the emergence of protons peaks attributed to methylene (–C_6_H_6_–CH_2_) and methane (–C_6_H_6_) were both observed at *δ* = 5.27 and *δ* = 7.38 ppm, respectively. The proton peaks assigned to methylene neighborhood to disulfide (OCCH_2_CH_2_–S–S) were located at *δ* = 2.76–2.99 ppm. After the Mal-PEG-ss-COOH conjugated on the PMSL blocks, the corresponding proton peaks attributed to PEG and PMSL blocks were detected in the spectrum of Fig. 1C, implying the successful introduction of modified PEG segment to PMSL blocks. Moreover, the chemical shifts of protons assigned to the HSPs were observed at *δ* = 6.4–7.0 ppm, *δ* = 8.3 ppm after being immobilized on Mal-PEG-ss-PMSL through Michael addition reaction. The above results indicated the successful synthesis of the target copolymer.

### Physicochemical properties of multifunctional polymeric micelles

**Figure 2:**
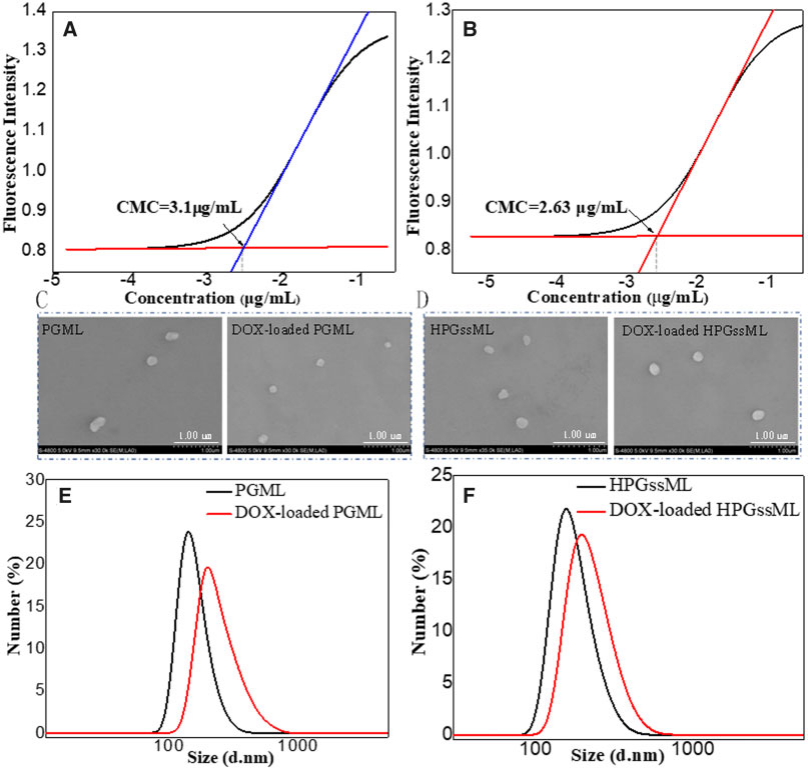
The CMCs of PGML (**A**) and HPGssML (**B**), the morphologies of blank and DOX-loaded PGML (**C**) and HPGssML (**D**), the size of blank and DOX-loaded PGML (**E**) and (**F**) HPGssML, respectively.

The synthesized multifunctional copolymers were amphiphiles, which can self-assemble into polymeric micelles in aqueous solution with the aptamer-decorated PEG segment as the hydrophilic shell and the polyester segment with π-conjugated moieties as the hydrophobic core. The micellar formation process was evaluated by CMC, which is an indicator of the stability of polymeric micelles. The CMC values of PGML and HPGssML polymeric micelles were 3.10 and 2.63 μg/mL, respectively (Fig. 2 and Supplementary Table S1), which was lower than that of conventional PEG-PCL polymeric micelles, supporting that the π-conjugated architectures in polymers can improve their stability [33].

**Figure 3:**
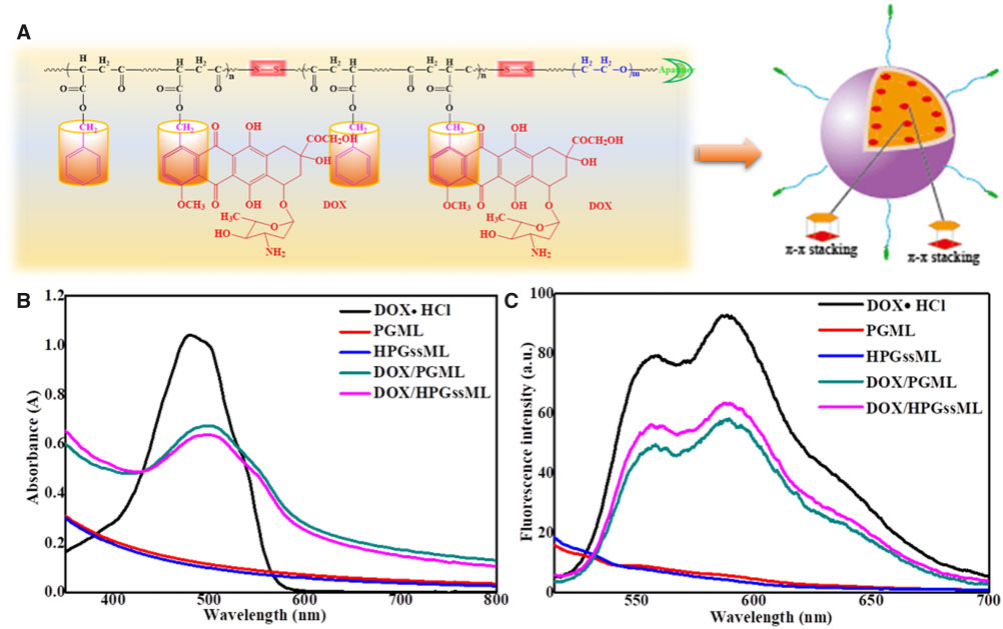
The schematic of self-assembly process of polymeric micelles via π–π interaction (**A**), UV-vis spectra of micelles in aqueous solution (**B**), fluorescence spectra of micelles in aqueous solution at 485 nm excitation wavelength (**C**), the concentration of DOX was 60 µg/mL.

The average size of both polymer micelles was about 150 nm with narrow size distributions of 0.3 (Fig. 2E, F and Supplementary Table S1). After DOX loading into polymeric micelles, particle sizes increased by DLS detection, implying DOX entrapment in polymeric micelles. Scanning electron microscopy images presented spherical nanostructures with a diameter of ∼200 nm for both polymeric micelles (Fig. 2C, D), which were in good agreement with DLS results. The maximum loading capacity of DOX reached up to 18%, which was higher than that of conventional polymeric micelles, such as mPEG-PLLA, mPEG-PCL polymeric micelles [5, 34]. For example, Cheng *et al*. reported that the DLC of polymeric micelles self-assembled by mPEG5k-b-PLLA5k and mPEG5k-b-PCL5k were 3.8 and 5.1%, respectively, due to the lack of π–π structures in polymers [34]. But, when the small molecules with π–π-conjugated structures were introduced into mPEG-2K-[G2]-CIN polymer, the enhancement of DLC (∼15.7%) was obtained [5]. The improved drug loading capacity for the constructed polymeric micelles was derived from the strong π–π stacking interaction between the hydrophobic core and the drugs, which was also in keeping with our previously reported results [5]. To explore the intrinsic essence of the DLC improvement in polymeric micelles, fluorescence spectra and UV-vis absorption were employed to detect the π–π stacking interaction within DOX-loaded micelles. A remarkable decrease in fluorescence intensity with the same exciting wavelength and DOX concentration was observed, due to aggregation-caused DOX fluorescence quenching (Fig. 3), suggesting the formation of compact polymeric micelles with π–π stacking interactions. The UV absorbance of DOX-loaded micelles offered the same evidence of the formation of π–π stacking interactions in polymeric micelles. Fig. 3B showed that no absorbance in the range of 350–700 nm was observed in the blank micelles, whereas free DOX exhibited significant absorbance at 480 nm. After being loaded with DOX, an obvious red shift (∼498 nm) was detected in the absorption peaks of DOX. The generation of the redshift of DOX was attributed to the ground-state electron donor–acceptor interaction or π–π stacking between the two parts. This phenomenon was good according to π–π stacking behaviors of π-conjugated modified polymeric assemblies [7, 17].

### Reduction-responsive behaviors of polymeric micelles

**Figure 4:**
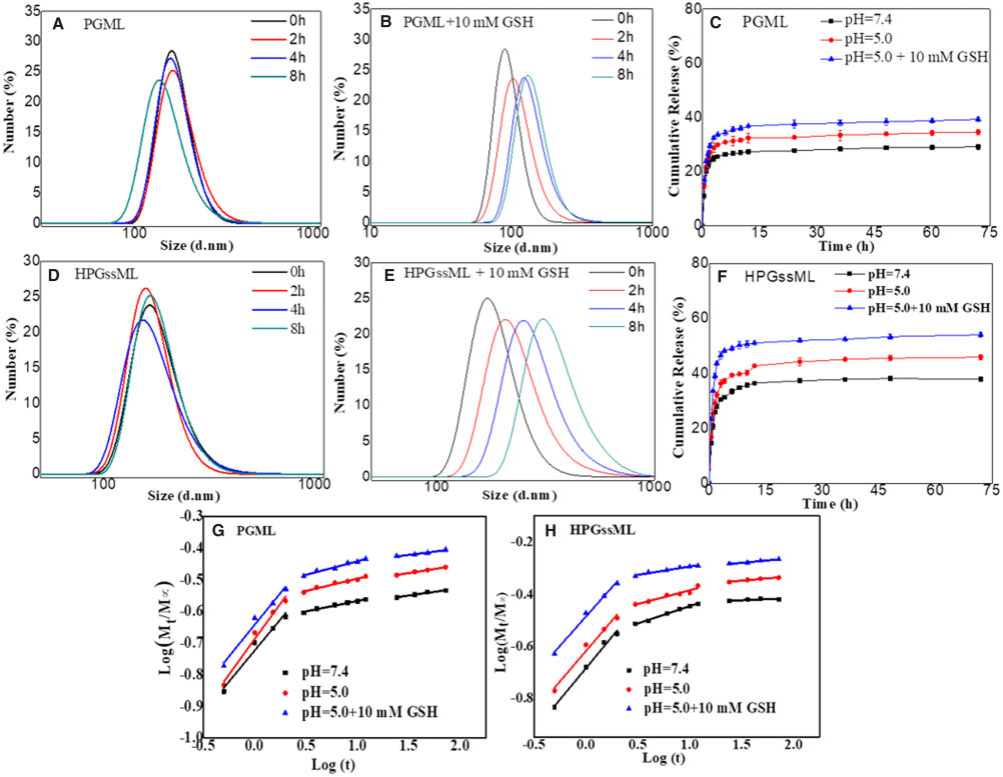
The size changes of (**A**, **D**) PGML and (**B**, **E**) HPGssML in different concentrations of GSH solutions. The release profiles of DOX from DOX-loaded micelles in different conditions at 37°C *in vitro* from (**C**) PGML and (**F**) HPGssML. The results are expressed as mean ± SD (*n* = 3). Plots of log(*Mt*/*M∞*) against log*t* for DOX release from (**G**) PGML and (**H**) HPGssML.

The reduction-sensitive behaviors of polymeric micelles were monitored by size change and *in vitro* drug release within the reductive stimulated environment. As shown in Fig. 4, the particle size of HPGssML significantly increased from 150 to 700 nm following treatment with 10 mM GSH, corresponding to intracellular redox condition. This suggested that GSH triggered the breakage of reductive–sensitive linkage. For the PGML group, a small size change should be attributed to the cleavage of disulfide linkage in the core of polymeric micelles, which would not destroy the micellar structure because of the PGML copolymer with the amphiphilic properties. Interestingly, the redox conditions (10 mM GSH) did not dissociate the micellar structure of HPGssML polymeric micelles, possibly due to the existence of strong π–π interaction in the core of the micelles. Meanwhile, in the absence of GSH, the particle sizes of polymeric micelles varied little at various intervals, indicating good stability of polymeric micelles.

The drug release behaviors in different released media also provided powerful evidence that the cleavage of the disulfide bond in polymeric micelles prompted faster drug release. As shown in Fig. 4C, F, at physiological condition (pH 7.4), <29% and 38% of DOX was released from the micelles after 72 h, respectively, revealing that polymeric micelles could steadily retain drugs in neutral medium. On the contrary, the amount of DOX released from both polymeric micelles could go up to about 35% and 46% at pH 5.0, respectively. At low pH value, protonation of DOX increased its solubility in water, resulting in easier diffusion from polymeric micelles. Obviously, the presence of 10 mM GSH at pH 5.0 can induce the fast release of DOX due to the breakage of the reductive–sensitive linker and protonation of DOX. Although the drug release was improved within the first 12 h, the release behavior presented as a sustainable profile until 72 h, and the final accumulative DOX release from HPGssML was only near to 60%. Similar phenomena were also observed in the PGML group. The sustainable release behavior should be attributed to the strong π–π stacking interaction between the hydrophobic core and the drugs, which restricted dissociation of the micellar core. The size change behaviors of both polymeric micelles in a reductive environment were in good accordance with the release behaviors. The mechanism of drug release was also evaluated by the classical equation: *M_t_*/*M*_∞ _=*kt^n^* [35–37]. The fitting curves and corresponding data (*k*, *n*) are exhibited in Supplementary Table S2 and Fig. 4G, H. Notably, all release processes were controlled by the superposition of diffusion and swelling release, as well as by Fickian diffusion-controlled release in the following two stages. Altogether, we successfully constructed reduction-sensitive polymeric micelles, which can efficiently load an anticancer drug with strong π–π stacking and release the drug in a stimulus-responsive way.

### Intracellular internalization efficacy

**Figure 5:**
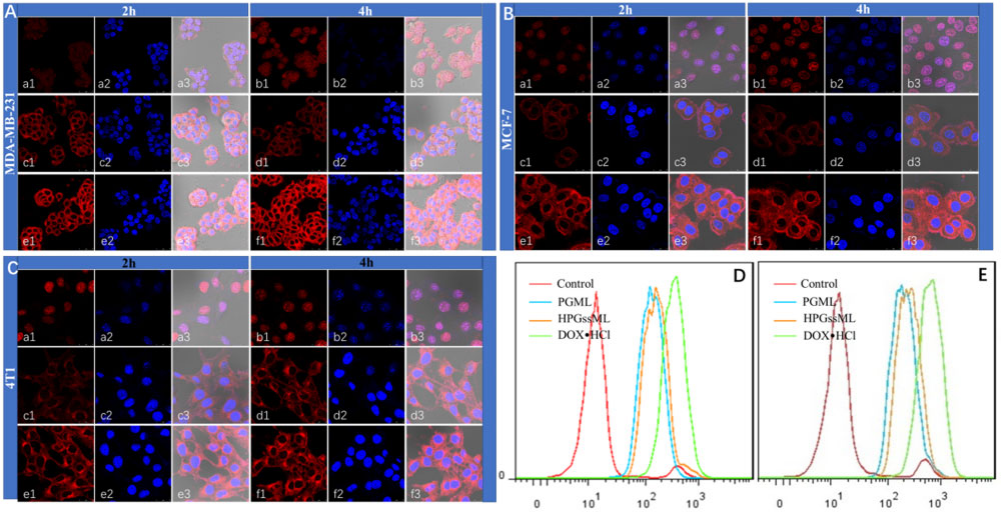
The confocal laser scanning microscopy images of MCF-7 cells (**A**), MDA-MB-231 (**B**) and 4T1 (**C**) cells treated with DOX-loaded nanoparticles. DOX · HCl (a, b), DOX-loaded PGML (c, d) and DOX-loaded HPGssML (e, f) for 2 and 4 h. The flow cytometry results of MCF-7 cells treated with DOX-loaded micelles for 2 h (**D**) and 4 h (**E**). The DOX concentration was 10 μg/mL. The flow cytometry results of MCF-7 cells treated with DOX-loaded micelles for 2 h (bottom left) and 4 h (bottom right), and the DOX concentration was 10 μg/mL.

The intracellular internalization of polymeric micelles was detected by confocal laser scanning microscopy and flow cytometry. In line with some previous reports on DOX · HCl internalization efficacy, among 4T1, MCF-7 and MDA-MB-231 cells, the strongest red fluorescence in the nucleus was observed in the DOX · HCl group for 2 and 4 h. Although DOX-loaded PGML and DOX-loaded HPGssML can be internalized into cells, most of the red fluorescence was located in the cytoplasm. As incubation time was prolonged, the red fluorescence in all cell types became stronger, indicating that more DOX-loaded micelles were internalized into cells. More importantly, as shown in Fig. 5, stronger red fluorescence intensity was observed in DOX-loaded HPGssML compared with DOX-loaded PGML for all cell types, indicating polymeric micelles can be internalized into cells via receptor-mediated endocytosis. The corresponding phenomena were further verified by quantitative evaluation obtained by flow cytometry (Fig. 5D, E). Collectively, the improvement of internalization efficiency for polymeric micelles can be attributed to the tumor homing ability of aptamer.

### *In vitro* cytotoxicity evaluation

**Figure 6:**
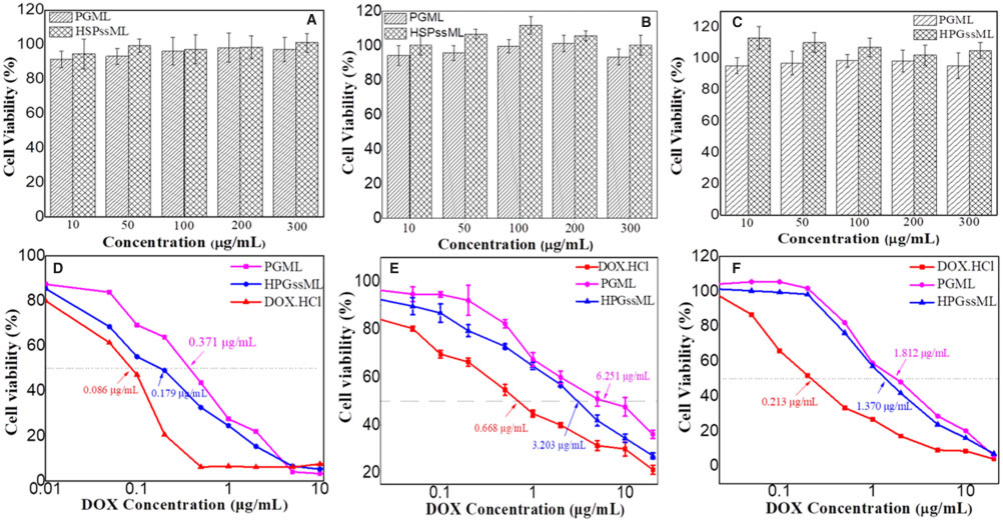
Cytotoxicity of PGML and HPGssML against (**A**) L929 cells, (**B**) MDA-MB-231 cells and (**C**) 4T1 cells for 48 h. The anticancer activity of DOX ⋅ HCl, DOX-loaded micelles against (**D**) MCF-7 (**E**) MDA-MB-231 cells (**F**) 4T1 cells *in vitro*. The results are expressed as mean ± SD (*n* = 5).

Cell biocompatibility of polymeric micelles against healthy and tumor cells was investigated by MTT assays. As shown in Fig. 6A–C, relative cell viability of polymeric micelles in L929, MDA-MB-231 and 4T1 cells were over 90%, even as the concentration of polymeric micelles was up to 300 μg/mL. These results indicated that the designed polymeric micelles were nontoxic to healthy and cancer cells. After loading with DOX, *in vitro* anticancer activity of DOX-loaded PGML and HPGssML was evaluated against cancer cells. 4T1, MDA-MB-231 and MCF-7 cells expressing HSP70 were chosen to explore tumor-targeting ability. Results in Fig. 6D–F and Supplementary Table S3 showed that the antitumor efficiency of both free DOX · HCl and DOX-loaded polymeric micelles all depended on DOX concentration. For 4T1 cells, the half-maximal inhibitory concentration (IC_50_) values of the DOX · HCl, DOX-loaded PGML and DOX-loaded HPGssML were 0.213, 1.812 and 1.370 μg/mL, respectively. Meanwhile, for MDA-MB-231 cells and MCF-7 cells, the IC_50_ values were 0.668, 6.251, 3.203 μg/mL and 0.086, 0.371, 0.179 μg/mL, respectively. Obviously, the best anticancer efficiency was observed in the DOX · HCl group because of its water solubility, allowing the induction of cell death after diffusion into cells. Additionally, the IC_50_ values of DOX-loaded HPGssML against the three cancer cells were all lower than the DOX-loaded PGML. The enhanced antitumor efficacy indicated the importance of active-targeting delivery DOX to cells and redox-sensitive release DOX in the cytoplasm for killing cancer cells.

## Conclusions

In summary, we have developed active-targeting, redox-activated biodegradable polymeric micelles with high-drug-loading capacity for cancer therapy. This type of polymeric micelle possessed good stability and high DLC, due to the strong π–π interaction between DOX and π-conjugated moieties in hydrophobic blocks. The variation of the micellar structure in a reductive environment induced the fast release of DOX from polymeric micelles. More importantly, the immobilization of aptamer on the surface of polymeric micelles can actively guide the polymeric micelles internalized into cancer cells, and the breakage of the reductive-sensitive linker in polymeric micelles under a high concentration of GSH in tumor cells can accelerate the release of DOX into cancer cells, followed by killing the cancer cells with prominent antitumor efficacy. We believe that our strategy is highly valuable for developing multifunctional biodegradable nanocarriers and achieving advanced therapeutic efficacy.

## Supplementary data

Supplementary data are available at *REGBIO* online.

## Supplementary Material

rbaa027_Supplementary_Data
