# Supplementary material for: High-drug-loading capacity of redox-activated biodegradable nanoplatform for active targeted delivery of chemotherapeutic drugs
Source: Regen Biomater. 2020 Jul 6;7(4):359–69. doi: 10.1093/rb/rbaa027 (PMC7414993; doi:10.1093/rb/rbaa027)
Supplement: rbaa027_Supplementary_Data [file rbaa027_supplementary_data.zip › rbaa027-Suppl_Data/Revised Supporting Information.docx]

***Supporting Information***

**High Drug Loading Capacity of Redox-activated Biodegradable Nanoplatform for Active Targeted Delivery of Chemotherapeutic Drugs**

Hai Zhang^a, b, #^, Jianqin Yan ^b, #^, Heng Mei ^b^, Shengsheng Cai ^b^, Sai Li ^a,^*, Furong Cheng ^b^, Jun Cao ^b,^*, Bin He ^b^

*^a^ School of Chemical Engineering, Sichuan University, Chengdu 610065, China*

*^b^National Engineering Research Center for Biomaterials, Sichuan University, Chengdu 610064, China.*

Corresponding authors: lisai@scu.edu.cn (S.L), caojun@scu.edu.cn (J.C)

*Fax: +86-28-85412923; Tel: +86-28-85470770*

^#^ These authors contributed equally to this work.

*Synthesis of* *Benzyl Malolactonate Monomer (BMA).* The synthesis of benzyl malolactonate monomer (BMA) was according to the procedure previously reported. Firstly, (R)-bromosuccinic acid (15 g, 76.1 mmol) was put into round bottom flask and vacuum-dried for 6 h. Then 45 mL of anhydrous THF was added and stirred under nitrogen atmosphere. Trifluoroacetic anhydride (19.2 g, 91.4 mmol) was added into the mixture in an ice bath and stirred for 4 h. The solution was condensed and benzyl alcohol (9.05 g, 83.8 mmol) was added for stirring another 12 h in an oil bath at 45 ℃. After that, the product was dissolved in diethyl ether and washed with deionized water. The organic phase was dried and decolored with anhydrous magnesium sulfate and activated carbon. The solution was filtered and the filtrate was condensed into yellow oily liquid.

The yellow oily liquid was transfer into round bottom flask and deionized water was added under the condition of vigorously stirring. The pH value of the solution was adjusted to 7.4-7.2 with 2 mol/L of NaOH. Then dichloromethane (CH_2_Cl_2_) was added into the mixture and refluxed for 4 h in an oil bath at 45 ℃. The organic phase was condensed and washed to neutral with deionized water and NaCl solution. The organic phase was dried with anhydrous magnesium sulfate, filtered with buchner funnel, condensed and dried. Finally, the crude product of pale-yellow liquid was obtained and purified using silicone gel column.

*Synthesis of mPEG-ss-COOH.* First，mPEG-OH（10 g, 5 mmol）and succinic anhydride (0.6 g, 6 mmol) was dissolved in trichloromethane (CHCl_3_). Then the mixture refluxed for 48 h in an oil bath at 75 ℃. After that，the mixture was condensed and precipitated in cold diethyl ether. The final white powder product was filtered and vacuum-dried at room temperature.

*Synthesis of* *PGML.* mPEG-COOH (0.38 g, 0.18 mmol), DMAP (2 mg, 0.018 mmol) and PMSL (0.68 g, 0.18 mmol) were dissolved in 20 mL anhydrous DCM in a bath under nitrogen atmosphere. DCC (0.745 g, 3.6 mmol) was dissolved in 20 mL DCM and added into the mixture. Then the solution was stirred at room temperature for 48 h. And the white precipitate of DCU (N, N'-dicyclohexylurea) was filtrated. The filtrate was condensed into 5 mL and precipitated in large cold diethyl ether. The crude product was vacuum-dried at room temperature.

**Scheme S1**. The synthetic routes of BMA, mPEG-COOH, Mal-PEG-OH, Mal-PEG-ss-COOH polymers.

**Scheme S2.** The synthetic routes of PGML copolymer.


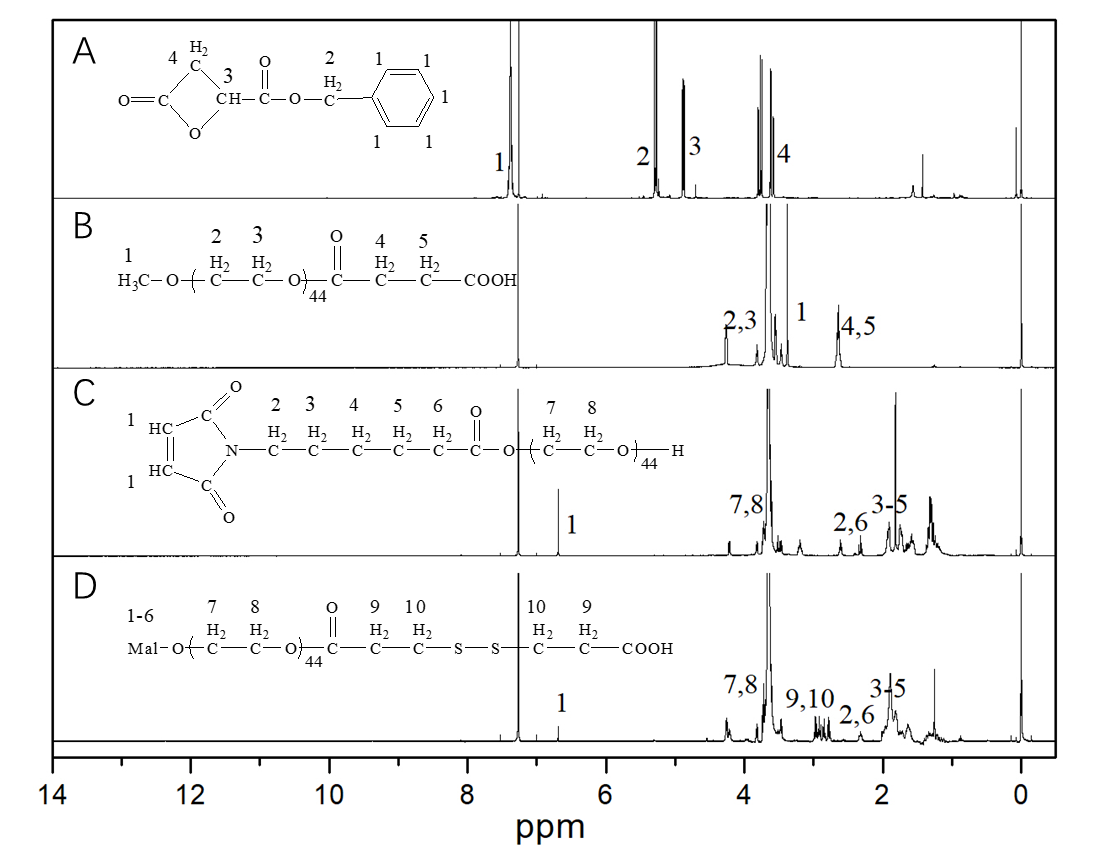


**Figure S1**. The ^1^H NMR spectra of (A) BMA, (B) mPEG-COOH, (C) Mal-PEG-OH, (D) Mal-PEG-ss-COOH in CDCl_3_.

**Table S1.** The properties of PGML and HPGssML polymeric micelles.

| Polymeric micelles | Blank |  | DOX-loaded | CMC  (μg/mL) | DLC  (%) | DLE |
| --- | --- | --- | --- | --- | --- | --- |
|  | Size (nm) PDI |  | Size (nm) PDI |  |  | (%) |
| PGML | 141 0.252 |  | 190 0.324 | 3.10 | 18.2 | 52.0 |
| HPGssML | 164 0.332 |  | 191 0.351 | 2.63 | 17.9 | 50.9 |

**Table S2.** Release exponent (n), rate constant (k), and correlation coefficient (R^2^) for the two drug-loaded micelles

| Samples | Time interval | pH=7.4 | | | pH=5.0 | | | pH=5.0+10 mM GSH | | |
| --- | --- | --- | --- | --- | --- | --- | --- | --- | --- | --- |
|  |  | n | k | R^2^ | n | k | R^2^ | n | k | R^2^ |
| PGML | 0-2h | 0393 | 0.135 | 0.960 | 0.447 | 0.206 | 0.973 | 0.400 | 0.228 | 0.973 |
|  | 3-12h | 0.066 | 0.233 | 0.979 | 0.078 | 0.267 | 0.963 | 0.086 | 0.297 | 0.977 |
|  | 24-72h | 0.046 | 0.240 | 0.970 | 0.052 | 0.277 | 0.996 | 0.041 | 0.329 | 0.945 |
| HPGssML | 0-2h | 0.482 | 0.206 | 0.987 | 0.466 | 0.240 | 0.970 | 0.449 | 0.326 | 0.989 |
|  | 3-12h | 0.133 | 0.263 | 0.993 | 0105 | 0.323 | 0.915 | 0.065 | 0.438 | 0.950 |
|  | 24-72h | 0.015 | 0.356 | 0.999 | 0.035 | 0.397 | 0.944 | 0.037 | 0.462 | 0.950 |

**Table S3** the IC_50_s of DOX-loaded polymeric micelles for MDA-MB-231 cells, 4T1 cells and MCF-7 cells.

| polymeric micelles | DOX·HCl  (μg/mL) | DOX/PGML  (μg/mL) | DOX/HPGssML  (μg/mL) |
| --- | --- | --- | --- |
| MDA-MB-231 | 0.608 | 6.251 | 3.203 |
| 4T1 | 0.213 | 1.812 | 1.370 |
| MCF-7 | 0.086 | 0.371 | 0.179 |
