# Supplementary material for: High-drug-loading capacity of redox-activated biodegradable nanoplatform for active targeted delivery of chemotherapeutic drugs
Source: Regen Biomater. 2020 Jul 6;7(4):359–69. doi: 10.1093/rb/rbaa027 (PMC7414993; doi:10.1093/rb/rbaa027)
Supplement: rbaa027_Supplementary_Data [file rbaa027_supplementary_data.zip › rbaa027-Suppl_Data/Point-by-Point Response Reviewer Comments.docx]

**Dear Dr. Peppas,**

Thank you very much for providing us this opportunity to revise our manuscript (Manuscript ID: No. RB-2020-036). We have seriously considered and point-by-point addressed the reviewer’s remarks, and revised the manuscript accordingly. Also, we have made changes in format at requested by editorial office. We hope now that the highly improved manuscript has reached the standard of the journal for publication. All changes in details are listed in the following.

Comments to the Author

The manuscript by Hai Zhang and coworkers reported on active targeting, redox-activated polymeric micelles (HPGssML), which exhibited GSH-sensitive drug releasing character and high drug loading content (~18%) via strong π-π interaction. This study showed much promise as a potential strategy for drug delivering. Overall, I would like to recommend it to be accepted for publication in Regenerative Biomaterials after minor revisions as listed below:

1) Some images should be improved to help readers get information more quickly. For example, Figure 2(A, B), dotted line should be drawn from the turning point to the abscissa to show the CMC more clearly. Figure 2(C), the scale label should be placed observably.

**Authors’ reply:** Thank you very much for pointing out the shortcomings and we have now corrected them as shown in Figure 1.

**Figure 1.** The CMCs of PGML (A) and HPGssML (B), the morphologies of blank and DOX-loaded PGML (C) and HPGssML (D), the size of blank and DOX-loaded PGML (E) and HPGssML respectively.

2) Some sentences should be modified to ensure accurate representation.

**Authors’ reply:** Thanks a lot for reviewer’s suggestion. We have revised the whole manuscript by a native speaker.

**Reviewer: 2**

Comments to the Author

The manuscript by Cao and Li et al. describes the synthesis and characterization of new polymeric raw materials for DDS generation. The novel raw materials were designed to enhance drug loading by π-π stacking and improve cellular uptake by the incorporation of active-target ligands. Furthermore, redox-sensitive bonds were incorporated into the polymeric structure to encourage the release of drugs in tumour cells. Overall, the study is technologically and mechanistically sound, and the findings are matched with the designed purpose. It is recommended to publish in Regenerative Biomaterials after revision. There are a number of issues listed below that the authors should consider when revising their manuscript.

Comments:

1. The author synthetized new copolymers which were characterized by NMR. It is better to provide the number of PCL and PMA blocks in polymer through the peak areas from HNMR. The related solvents used in detection of NMR spectra for each polymer should be incorporated in the manuscript.

**Authors’ reply:** According to the integral area, the number of PMA and PCL blocks in the polymer are 7 and 15, respectively. The related solvents used in the detection of NMR spectra for each polymer have been incorporated in the manuscript.

2. It is suggested to add some more discussion in comparison with other works reported to support the high LE% claimed by the authors.

**Authors’ reply:** As the reviewer’s suggested, Some discussion has been added in comparision with other works reported to support the high DLE.

3. In Figure 4, what about the influence of DOX-loaded micelles on L929 cells?

**Authors’ reply:** In this study, the polymeric micelles without DOX influenced on the L929 cells were conducted and no cytotoxicity on L929 cells was observed, indicating good cell biocompatibility. DOX, as an anticancer agent, was mostly used to treat cancer.So, in this study, the influence of DOX-loaded micelles on cancer cells was conducted and results showed that the cancer cells can be efficienctly killed. Certaintly, if the DOX-loaded micelles were incubated with L929 cells, L929 cells can be killed due to the cytotoxicity from DOX. However, as the DOX loaded into polymeric micelles with nanosized character, they can be usually accumulated into tumor site through tumor homing effect and EPR effect, and little effects on the healthy cells, like L929. Therefore, the influence of DOX-loadedd micelles on L929 cells would not be conducted.

4. The author mentioned that 'as shown in Fig 3II(A, B), at physiological pH (7.4), less than 20% of the drug was released from the micelles after 72 h'. But from the figures, the cumulative release is both above 20%. The statement is wrong.

**Authors’ reply:** we edited on Page 12 “As shown in Fig 4(C, F), at physiological condition (pH 7.4), less than 29 % and 38 % of DOX was released from the micelles after 72 h, respectively, revealing that polymeric micelles could steadily retain drugs in neutral medium.”

5. About the redox-responsive behaviors and the drug release behaviors tests, why not to choose the same conditions of the biomimetic solutions?

**Authors’ reply:** Thanks a lot for the reviewer’s valuable suggestion. The redox-responsive behavior of HPGssML was detected, adopting a reductionist approach (10 mM GSH media as a biomimetic solution) to demonstrate the presence and activity of redox-sensitive disulfide bonds on HPGssML. In drug release behaviors tests, due to intra-tumor microenvironment with acid and high GSH, the drug release behaviors tests were conducted under pH5.0+10 mM GSH to mimic those conditions.

6. Some minor spelling mistakes and format in the manuscript are needed to be revised. For example, in the introduction section, “β-Benzyl ester malic acid lactone (b-MLABz) is a side protected malic acid lactone monomer.” should be revised as “β-Benzyl ester malic acid lactone (β-MLABz) is a side protected malic acid lactone monomer.” “For instance, the presence of a high level of glutathione (GSH, 2~10 mM)) in tumor cells”, the bracket could be deleted. In Scheme 1, the abbreviation “PLMBz-PCL” is not in accordance with that of in the manuscript. Also, other section, for example, in the Materials section, there are different abbreviations shown “HSP-PEG-ss-PMSL” and “HPGssML”. So, it should be verified those abbreviation.

**Authors’ reply:** We edited on Page 2 “β-Benzyl ester malic acid lactone (β-MLABz) is a side protected malic acid lactone monomer. It is generally used as a monomer in the synthesis of biodegradable poly(β-benzyl ester malate) (PMLABz) via ring-opening polymerization[18]. Most studies have focused on constructing intelligent polymeric micelles through conjugating various functional molecules with carboxyl groups on the side chains of poly(β-malic acid) (PMA) after deprotection[19, 20].” and on Page 3 “For instance, the presence of a high level of glutathione (GSH, 2~10 mM) in tumor cells, compared to that of normal tissues, proves that the disulfide bond can be easily cleaved by intracellular GSH while remaining stable in physicochemical conditions [31].”. We have corrected the abbreviations in Scheme 1. All the abbreviations have also been corrected.

**Scheme 1.** The aptamer-decorated, reduction-sensitive polymeric micelles self-assembled biodegradable polyester-based amphiphilic copolymer and the concept for a proposed behavior of polymeric micelles for anticancer drug loading, delivery and release.
